# Factors Associated with Burnout Among Nurses: The Roles of Shift Work Characteristics, Work–Life Balance Satisfaction, and Chronic Disease

**DOI:** 10.3390/healthcare14183015

**Published:** 2026-09-15

**Authors:** Enas Mohamed Lotfy, Ibrahim Naif Alenezi, Faisal Khalaf Alanazi, Safaa Gaber ALY Salem, Nawal Ali Abdelaziz Ammar, Manal Saleh Moustafa Saleh, Mohammed Alshmemri, Ohoud Naif Aldughmi

**Affiliations:** 1 Public Health Nursing Department, College of Nursing, Northern Border University, Arar 73213, Saudi Arabia; 2 Medical and Surgical Nursing Department, College of Nursing, Northern Border University, Arar 73213, Saudi Arabia; 3 School of Nursing, Midwifery & Paramedicine, Australian Catholic University, Banyo, QLD 4014, Australia; 4 Department of Maternity, Obst & Gyn Nursing, College of Nursing, Princess Nourah Bint Abdulrahman University, P.O. Box 84428, Riyadh 11671, Saudi Arabia; 5 Hospital and Nursing Management, Military Medical Academy, Cairo 11291, Egypt; 6 Nursing Administration, Faculty of Nursing, Zagazig University, Zagazig 44519, Egypt; 7 Department of Nursing Sciences, College of Applied Medical Science, Shaqra University, Shaqra 11961, Saudi Arabia; 8 Nursing Sciences and Research Department, Faculty of Nursing, Umm Al-Qura University, Makkah 21955, Saudi Arabia

**Keywords:** burnout, nurses, work–life balance, shift work, chronic disease, cross-sectional study

## Abstract

**Highlights:**

**What are the main findings?**

- Work–life balance satisfaction showed the strongest association with burnout among the variables examined.
- Chronic disease showed a borderline association with higher burnout, whereas shift duration was not independently associated after adjustment, although the very limited variation in shift patterns restricts what can be concluded from this.
- The final hierarchical regression model explained 43.9% of the variance in burnout scores.
- Moderate-to-severe burnout affected 70% of the participating nurses.

**What are the implications of the main findings?**

- Work–life balance deserves attention in burnout prevention alongside shift scheduling, not in place of it.
- Organizational strategies including predictable scheduling, adequate recovery time, and supportive staffing policies may reduce nurse burnout.
- Early screening and occupational health support should be considered for nurses living with chronic diseases.
- Future longitudinal multicenter studies are needed to confirm causal relationships and evaluate intervention effectiveness.

**Abstract:**

Background: Burnout is a major challenge to the ongoing viability of the nursing workforce. However, only a limited number of studies have integrated work shift characteristics, satisfaction with work–life balance, and chronic illness into the same model. Aim: To examine whether shift work patterns, satisfaction with work–life balance, and chronic illness are independently associated with burnout among nurses. Methods: Between March and May of 2025, an analytical cross-sectional study was conducted on 100 nurses in two Egyptian military hospitals. Burnout was measured with the 20-item Burnout Syndrome Assessment Scale (BOSAS; Cronbach’s α = 0.832 in the present sample). Unadjusted associations were examined using univariable linear regression with HC3 robust standard errors, and hierarchical multiple linear regression was used to identify factors independently associated with the total burnout score. Results: The reported prevalence of burnout was 30.0% mild, 57.0% moderate, and 13.0% severe. The mean total burnout score was 46.72 ± 11.55. The final model of the regression explained 43.9% of the variance in burnout. Work–life balance satisfaction showed the strongest association with burnout among the variables in the model (standardized β = −0.479, *p* < 0.001), with each one-category increase in satisfaction corresponding, on average, to a 4.49-point lower burnout score. Chronic illness showed a borderline association with higher burnout (B = 4.40, *p* = 0.0499). In addition, nurses aged 36–45 years had lower scores than those aged 18–25 years. Shift duration, gender, and physical activity were not independently associated with burnout, although the near-uniform shift patterns in this sample limited the ability to detect an effect of shift length. Conclusions: In this sample, greater work–life balance satisfaction was associated with lower burnout, while the near-uniformity of shift exposure limited what could be concluded about shift duration. The cross-sectional design precludes causal inference and the direction of these relationships cannot be established. Preventive measures that protect work–life balance, such as predictable rosters, sufficient recovery time, and staffing that eases competing work and family demands, warrant evaluation, as does early support for nurses living with chronic disease.

## 1. Introduction

Burnout is rapidly becoming a substantial occupational issue within the nursing workforce. Traditionally viewed as a multi-faceted response to prolonged exposure to unresolved work-related stress [1], burnout is recognised in the eleventh revision of the International Classification of Diseases as an occupational phenomenon arising from chronic workplace stress that has not been successfully managed [2]. The nature of nurses’ day-to-day work is particularly demanding because it combines sustained emotional demands, high workload, and time pressure with the continuous responsibility of patient care [3].

The magnitude of the issue is significant. A meta-analysis that covered 49 countries estimated that about one in every nine nurses worldwide is affected by burnout [4]. Recent meta-analyses show that the burden of nursing burnout has not diminished, and may be increasing, in a number of regions over the past ten years [5]. The COVID-19 pandemic worsened the emotional and mental fatigue crisis and increased the depersonalization of the nursing population [6]. The ramifications of burnout are not only personal; burnout has been related to a decline in patient safety and an increase in the intention to leave the nursing profession [7,8]. Against a background of nursing shortages in many health systems, understanding what drives burnout is therefore a question of workforce sustainability and not only of individual wellbeing.

Shift work is among the most frequently examined occupational exposures. In European survey data, shifts of 12 h or longer have been associated with greater emotional exhaustion, stronger intention to leave, and lower job satisfaction [9]. Longer weekly working hours, night work, and short recovery between shifts have likewise been linked to higher burnout among hospital nurses [10]. Additionally, the rotating schedules and sleep disturbances from working shifts have been associated with poorer mental health and increased burnout [11,12]. The evidence is nonetheless mixed, and the association between shift length and burnout has been inconsistent across settings.

Work–life balance has emerged as a further consistent correlate. Nurses who struggle to reconcile work and personal life report more burnout, less job satisfaction, and stronger intentions to reduce their hours or leave nursing [13]. Integrative and cohort evidence from nursing in the fields of oncology and hematology identifies poor work–life balance as a consistent factor associated with burnout [14,15]. It remains less clear whether dissatisfaction with work–life balance is associated with burnout independently of the shift patterns and personal characteristics with which it tends to co-occur.

Personal health may also be relevant. Emerging evidence suggests a bidirectional relationship between burnout and physical health: burnout may forecast future somatic illnesses, and poor physical health decreases the ability to manage persistent physical demands related to work [16]. Among specific healthcare professionals, the relationship between poorer physical and mental health and higher burnout levels is noted [17]. Meta-analytic evidence also links younger age with higher burnout [18]. Few studies, however, have modeled chronic disease together with shift work and work–life balance, so the contribution of chronic illness to nurse burnout, independent of these factors, remains uncertain.

Nurses’ burnout is frequently related to shift work characteristics, satisfaction with work–life balance, and chronic illness. However, within a single model adjusted for other variables, especially in Middle Eastern countries and in workforces subject to long shifts, these are rarely studied together. This study therefore examined whether these three factors were independently associated with burnout among nurses in two military hospitals in Egypt after accounting for demographic and health-related characteristics. Given the cross-sectional design, in which exposures and outcome were measured simultaneously, the findings are framed throughout as associations rather than as predictors in any temporal or causal sense.

## 2. Methods

### 2.1. Study Design and Setting

The study employed an analytical cross-sectional design and was reported in accordance with the Strengthening the Reporting of Observational Studies in Epidemiology (STROBE) guidelines (Supplementary Material). The study was conducted in two military hospitals in Egypt between March and May 2025, and nurses working across the hospitals’ clinical departments were included. This design allowed burnout, shift work characteristics, work–life balance satisfaction, chronic disease, and other participant characteristics to be assessed at the same time and their independent associations with burnout to be estimated. The data derive from a master’s thesis project conducted at the Military Medical Academy. Descriptive findings from the same cohort, focusing on the bivariate relationship between long working hours and burnout, have been reported previously [19]; that earlier report was confined to the demographic and work profile of the sample, the descriptive distribution of burnout levels, and the unadjusted correlation between long working hours and total burnout (r = 0.167, *p* = 0.097). It contains none of the univariable HC3 regressions, none of the hierarchical models, and none of the adjusted coefficients reported here. The present study addresses a different question, namely the identification of factors independently associated with burnout, through hierarchical multivariable modeling with adjustment for demographic, health-related, and occupational characteristics.

### 2.2. Participants and Sampling

Participants were recruited by convenience sampling from the clinical departments of the two hospitals. Recruitment covered the surgical, emergency, orthopaedic, paediatric, intensive care, labour, endoscopy, dialysis, medical, cardiac, and burns units, so the sample spans a wide range of clinical areas rather than a single specialty. All nurses working in the participating hospitals during the study period were eligible; nurses on leave during data collection were excluded. The final sample comprised 100 nurses who were present in their units during the data collection visits and who agreed to take part. The study records do not document the size of the total eligible nursing workforce, the number of nurses approached, or the number who declined, and we therefore do not report a conventional response rate. What can be stated is that all questionnaires distributed were returned complete, which most likely reflects the in-person distribution and collection within the clinical units. The implications of that procedure are considered in the limitations.

### 2.3. Sample Size

An a priori calculation was originally performed for a comparison of mean burnout scores between two groups, using the standard formula for comparing two means, *n* = 2(Zα/2 + Zβ)^2^σ^2^/d^2^, with a two-sided α of 0.05 (Zα/2 = 1.96), power of 80% (Zβ = 0.84), an assumed standard deviation of 10.7, and a minimum detectable difference of 4.2 points. Under these assumptions the formula yields approximately 100 participants per group, or about 200 in total, and it addresses a two-group comparison rather than the multivariable analysis reported here. In practice, the achieved sample of 100 nurses was determined primarily by the number of eligible nurses available in the two hospitals during the study period, and we state this openly rather than presenting the calculation as the basis of the final sample. The final sample for analysis consisted of 100 nurses with complete questionnaire data. In light of the sample size available for this study, the multivariable model was intentionally limited to a small set of theoretically relevant variables so as to avoid overfitting and instability of the regression estimates. Shift duration, work–life balance satisfaction, and chronic disease were the primary explanatory variables, with age, gender, and physical activity included for adjustment. The resulting model contained eight estimated parameters, or approximately 12.5 participants per parameter. We regard this ratio as a safeguard against overfitting rather than as formal evidence of adequate power, and the possibility that modest associations went undetected is acknowledged in the limitations.

### 2.4. Measures

Participant, work, and health characteristics: A structured self-administered questionnaire collected data on age, sex, marital status, educational qualification, years of professional experience, current position, clinical department, and place of residence. Work-related and health variables covered shift type, average weekly working hours, number of night shifts per month, employment status, physical activity level, presence of chronic disease, and satisfaction with work–life balance. Work–life balance satisfaction was assessed with a single ordinal item offering five response categories from very satisfied to very dissatisfied, and chronic disease was recorded as the presence or absence of any self-reported chronic health condition.

**Burnout Syndrome Assessment Scale:** Burnout was measured with the Burnout Syndrome Assessment Scale (BOSAS). Unlike generic burnout measures such as the Copenhagen Burnout Inventory [20], the BOSAS is a 20-item instrument developed and validated specifically among nurses working in intensive care units [21], and its psychometric performance has since been examined in independent work on a translated version [22]. Although the instrument originated in intensive care, its items address sustained emotional and occupational demands that are common to hospital nursing rather than exposures unique to critical care, and it was therefore considered applicable to the nurses recruited here from a range of clinical departments; the absence of formal validation of the Arabic version across departments is nonetheless acknowledged as a limitation. In the present study the items were organized into two 10-item domains, personal burnout and occupational burnout, for descriptive purposes only. This grouping was adopted a priori, was not derived from a factor analysis of the present data, and all inferential analyses were conducted on the total score. Each item was rated on a five-point Likert scale from 0 (never) to 4 (always). Positively worded items were reverse scored before the domain and total scores were calculated, so that higher scores consistently reflected greater burnout. Each domain score ranged from 0 to 40 and the total score from 0 to 80. Total scores were classified as no burnout (0–20), mild burnout (21–40), moderate burnout (41–60), or severe burnout (61–80). The original instrument showed strong psychometric properties, including a Cronbach’s alpha of 0.94, test–retest reliability of 0.93, content validity index of 0.93, and concurrent criterion validity of 0.82 [21]. Internal consistency in the present sample was evaluated using Cronbach’s alpha.

### 2.5. Validity and Reliability

The BOSAS was translated from English into Arabic through a standardized forward–backward procedure. The Arabic translation was produced by a team of bilingual specialists, and an independent back-translation was done. The original and back-translated English versions were compared, and discrepancies were adjudicated by a panel of specialists in order to ensure linguistic clarity and conceptual equivalence. A pilot study with ten nurses examined completion time, feasibility, and clarity of wording. No substantive changes were required, and these participants were retained in the main sample. Beyond internal consistency, no psychometric evaluation of the Arabic version was undertaken in the target population, and the two-domain structure was not examined through factor analysis; the inferential analyses therefore rest on the total score.

### 2.6. Data Collection Procedure

From March to May 2025, self-administered questionnaires were used to collect data. The researcher visited the participating hospitals three times per week. During these visits, the researcher explained the purpose of the study to nurses, invited them to take part, and obtained their informed consent. The nurses filled out the questionnaires in their clinical units, and this was estimated to take around 15 to 30 min. Completed questionnaires were handed back within the clinical units and reviewed for completeness at the point of return; when items had been left blank, participants were asked whether they wished to complete them before submission. Because questionnaires were distributed and completed during working hours within the clinical units, the potential implications of this procedure for perceived voluntariness and response behavior are considered in the limitations.

### 2.7. Statistical Analysis

Categorical variables were summarized as frequencies and percentages, and continuous variables as means and standard deviations. Unadjusted associations between participant characteristics and the total burnout score were examined with univariable linear regression using HC3 heteroscedasticity-consistent robust standard errors; overall Wald tests were applied to variables with more than two categories, and work–life balance satisfaction was entered as an ordinal trend variable. Factors independently associated with burnout were then identified through hierarchical multiple linear regression built in three blocks: demographic characteristics (age and gender) in the first block; shift duration, chronic disease, and physical activity in the second; and work–life balance satisfaction in the third. Categorical explanatory variables were entered as dummy variables, with the following reference categories: age 18–25 years, male gender, 12-h shift, absence of chronic disease, and physical inactivity; regression coefficients therefore express the adjusted difference in the total burnout score relative to these reference groups. Work–life balance satisfaction was entered as a single ordinal trend variable coded from 0 (very dissatisfied) to 4 (very satisfied) rather than as dummy variables. Treating this item as a linear trend assumes approximately equal spacing between adjacent response categories; the implications of this assumption are considered in the limitations. As a sensitivity analysis, the final model was re-estimated with work–life balance satisfaction entered as dummy-coded categories rather than as a linear trend, to examine whether the association followed a monotonic gradient across the five response categories. We excluded average weekly hours from the model because weekly working hours were recorded only as ≥51 h for all participants; no finer-grained weekly hours measure was available in the study dataset. Night-shift frequency was likewise excluded because it applied only to the small subgroup of nurses working 12-h shifts.

To evaluate the assumptions of linear regression, the distribution and homoscedasticity of the residual, multicollinearity based on variance inflation factors, and influential observation based on Cook’s distance were examined. Unstandardized, robust, and standardized coefficients as well as 95% confidence intervals and *p* values have been reported. Model fit has been summarized using R^2^ and adjusted R^2^. A two-sided *p* value below 0.05 was considered statistically significant, and this convention was applied consistently across all tables; *p* values are reported to three decimal places, except where an additional decimal place is needed to resolve rounding at the significance threshold. Analyses were performed in IBM SPSS Statistics version 27.0.1.0 (IBM Corp., Armonk, NY, USA).

### 2.8. Ethical Considerations

Before the data collection, ethical approval was secured from the Military Medical Academy (IRB-MMA, approval No. 38-2024, dated 25 May 2024), and it was carried out in compliance with the Declaration of Helsinki. The directors of the participating hospitals gave administrative permission. Eligible nurses were notified of the study’s purpose and procedures, made aware of the voluntary nature of participation, the confidentiality of their responses and the right to withdraw from the study at any time without penalty. Written informed consent was obtained from all participants before questionnaire completion. No identifying information was collected, and the data were used exclusively for research purposes.

## 3. Results

### 3.1. Participant Characteristics

Table 1 summarizes the sociodemographic, occupational, and health characteristics of the participants. Most nurses were aged 18–35 years (94.0%), female (86.0%), and employed as staff nurses (89.0%). Eighty-seven percent worked 24-h shifts, and all participants reported working at least 51 h per week. Chronic disease was reported by 21.0% of the sample, and 43.0% described themselves as dissatisfied or very dissatisfied with their work–life balance.

### 3.2. Burnout Scores and Levels

The mean total BOSAS score was 46.72 ± 11.55, with mean personal and occupational burnout scores of 25.27 ± 6.54 and 21.45 ± 5.99, respectively (Table 2). More than half of the participants experienced moderate burnout (57.0%), 13.0% had severe burnout, and 30.0% had mild burnout; not a single participant fell into the no-burnout category. The total BOSAS demonstrated good internal consistency (Cronbach’s α = 0.832).

### 3.3. Unadjusted Associations with Burnout

In the unadjusted analyses (Table 3), total burnout scores differed significantly by age, chronic disease status, physical activity, and work–life balance satisfaction. Nurses with chronic disease scored higher than those without, burnout was greatest among physically inactive nurses, and scores fell progressively as work–life balance satisfaction increased. The association between shift duration and burnout approached but did not reach statistical significance; notably, mean burnout was higher in the small group working 12-h shifts than among those working 24-h shifts.

### 3.4. Hierarchical Regression Models

Demographic characteristics alone explained 9.4% of the variance in total burnout scores (Table 4). Adding shift duration, chronic disease, and physical activity raised the explained variance by 17.4 percentage points. Work–life balance satisfaction contributed a further 17.1 points, giving a final model that accounted for 43.9% of the variance in burnout scores.

### 3.5. Factors Independently Associated with Burnout

The final regression model was statistically significant and explained 43.9% of the variance in total burnout scores (Table 5). Work–life balance satisfaction showed the strongest association with burnout among the variables in the model (standardized β = −0.479, *p* < 0.001); under the linear-trend coding, each one-category increase in satisfaction corresponded, on average, to a 4.49-point lower total burnout score after adjustment for the other variables, though the unadjusted category means indicate that the gradient is uneven. Nurses aged 36–45 years had lower burnout scores than those aged 18–25 years (*p* = 0.039); because this subgroup comprised only six participants, the estimate is imprecise and should be regarded as exploratory. The presence of chronic disease was associated with an estimated 4.40-point higher burnout score at the margin of statistical significance (*p* = 0.0499), a result interpreted as suggestive rather than conclusive. Shift duration, gender, and physical activity were not independently associated with burnout in the adjusted model. The sensitivity analysis, in which work–life balance satisfaction was entered as dummy-coded categories with “very dissatisfied” as the reference, gave adjusted coefficients of B = −6.45 for dissatisfied (*p* = 0.046), B = −13.34 for neutral (*p* < 0.001), B = −13.32 for satisfied (*p* < 0.001), and B = −17.98 for very satisfied (*p* = 0.001). Burnout scores were consistently lower at every level above the reference, so the direction of the association was robust to the coding. The gradient, however, is not strictly monotonic: the neutral and satisfied categories differ by 0.02 points, and the largest single step falls between dissatisfied and neutral. The linear-trend coefficient of −4.49 should therefore be read as an average across an uneven gradient rather than as a constant decrement per category.

## 4. Discussion

### 4.1. Principal Findings

This study examined which factors were independently associated with burnout among nurses when shift work characteristics, work–life balance satisfaction, and chronic disease were considered together. Work–life balance satisfaction showed the strongest and most consistent association: nurses who were more satisfied with their work–life balance reported substantially lower burnout scores after adjustment for demographic, health-related, and shift-related characteristics. Chronic disease showed a borderline association with higher burnout. After adjustment, only the 36–45-year age group differed from the youngest nurses, and this estimate rests on a small subgroup and warrants caution. Shift duration, gender, and physical activity were not independently associated with burnout. The final model accounted for 43.9% of the variation in burnout scores, which suggests that individual health and occupational characteristics are worth modelling together with work–life balance rather than separately.

### 4.2. Burnout Burden in Context

Every participant scored above the no-burnout cut-off, and seven in ten were classified as experiencing moderate or severe burnout. This is far higher than the global prevalence of roughly one in nine nurses reported in meta-analytic research [4,5], yet the two figures cannot be compared directly, because prevalence estimates vary with the instrument, cut-off points, population, and operational definition of burnout used in each study. Part of the explanation may also lie in the classification thresholds of the BOSAS itself: with twenty items scored from 0 to 4, a respondent selecting the midpoint throughout would obtain a score of 40 and already fall within the mild-burnout range, and the lowest score observed in this sample (21) sits exactly at the lower cut-off. The 100% figure may therefore partly reflect the scoring structure of the scale rather than the work setting alone. Similar patterns of burnout have been shown in nursing workforces experiencing continuous excessive pressure. This has especially been the case during and after the COVID-19 pandemic [6]. The working conditions of this sample also matter: every participant exceeded 50 h a week, and most worked 24-h shifts. Neither explanation excludes the other. What follows is that the absolute prevalence reported here carries less weight than the relative differences between groups, and it should not be quoted alongside figures derived from other instruments.

### 4.3. Work–Life Balance Satisfaction

Nurses who reported greater satisfaction with the balance between their personal and professional lives had lower burnout scores, and this association persisted after demographic, health-related, and occupational factors were taken into account. This accords with earlier work linking poor work–life integration to lower job satisfaction, higher burnout, and stronger intentions to cut hours or leave nursing altogether [13,14,15]. Long and irregular hours, low recovery time, and competing family obligations allow work to encroach steadily on personal time, which in turn sustains cycles of mental and physical fatigue [13,14]. Two reservations qualify the finding. The cross-sectional design leaves the direction of the relationship open. Dissatisfaction with work–life balance may contribute to burnout, but nurses who are already exhausted may also come to judge their work–life balance more harshly, and the two processes are not mutually exclusive. There is a second and less obvious problem. Work–life balance and burnout were rated by the same people on the same occasion, so part of their association may be common-method and affective variance rather than a substantive relationship. That concern is distinct from the single-item issue and is not resolved by better measurement of the construct alone. Read with both reservations in place, work–life balance is best described as a promising and potentially modifiable organizational target, not as a demonstrated cause. Flexible schedules, predictable rosters, protected rest, and adequate staffing are plausible avenues for intervention, but none of them was evaluated here.

### 4.4. Chronic Disease

In the unadjusted analysis, nurses with chronic disease reported higher burnout; the association weakened after adjustment and rested at the margin of statistical significance (*p* = 0.0499). It is therefore treated as suggestive rather than as an established independent effect. Chronic morbidity among full-time nurses often develops where a physically and emotionally demanding job converges with limited opportunity for recovery and with health behaviors that deteriorate under such schedules [17]. One plausible reading is that chronic conditions deplete the reserves needed to meet persistent work demands, raising the effort each shift requires; the reverse is equally plausible, as prospective evidence indicates that burnout predicts the onset of new chronic disease [16]. The relationship is thus likely bidirectional, and only longitudinal research can disentangle it.

### 4.5. Shift Duration

Shift duration was not independently associated with burnout once demographic, health-related, and work–life balance characteristics were considered. At first glance this seems to contradict multicountry evidence linking shifts of 12 h or more to greater burnout [9,10]; two features of the present sample likely explain the difference. First, variability in exposure was minimal: 87% of nurses worked 24-h shifts and every participant exceeded 50 working hours per week, leaving little contrast against which an effect of shift length could be detected. Second, work–life balance satisfaction may lie on the causal pathway between shift patterns and burnout: long shifts erode recovery and personal time, which worsens perceived balance and in turn fosters burnout [11,12]. Where a variable functions as a mediator in this way, adjusting for it as a covariate can attenuate or obscure the association of the upstream exposure, so the adjusted coefficient for shift duration is best read as its association with burnout net of any effect transmitted through work–life balance, not as its total effect. For both reasons, the nonsignificant coefficient cannot be taken as evidence that shift length is unimportant for burnout. A further observation deserves comment: the small group of nurses working 12-h shifts reported a higher unadjusted mean burnout score than those working 24-h shifts. Given the marked imbalance between the two groups, this counterintuitive pattern most likely reflects sampling variability or unmeasured differences in workload rather than any protective effect of longer shifts.

### 4.6. Age, Gender, and Physical Activity

Burnout reports for nurses aged 18–25 were higher than those for nurses aged 36–45. A younger nursing workforce is known to exhibit higher emotional exhaustion and depersonalization, with support from recent meta-analytical work [18]. Early-career nurses must master demanding clinical skills while working schedules that leave limited room for recovery, which may constrain their coping resources. Selective attrition may also play a part, if nurses most affected by burnout leave the profession before reaching mid-career. This remains speculation; a cross-sectional sample of serving nurses cannot show who has already left. Because only six participants were aged 36–45 years, the estimate for this group is imprecise and should be viewed as exploratory rather than as evidence of a robust age effect.

Gender was not associated with burnout in this sample. The wider literature suggests that gender differences in burnout are modest and inconsistent, varying with professional role, work context, and the burnout dimension examined [23,24,25]. With men constituting only 14% of participants, this study had little capacity to detect such differences, and the null finding should be read accordingly. Regarding the relation of burnout and physical activity, there was an unadjusted association showing a gradient with the most physically inactive nurses reporting the most burnout. This gradient did not persist after adjustment. Physically active nurses generally report less burnout [26,27], and better recovery experiences among active nurses may partly account for that pattern [28]; the reverse direction is equally plausible, however, with exhausted nurses lacking the capacity for regular activity.

### 4.7. Final Interpretation and Implications

Within this sample, burnout was more closely associated with nurses’ perceptions of their work–life balance than with the nominal length of their shifts, although the restricted variation in shift patterns means the two exposures could not be compared on equal terms. For managers, this suggests that attention should extend beyond shift length alone to the predictability of schedules, opportunities for recovery, staffing flexibility, and managerial support. Systematic reviews indicate that structured support programs and workplace-level interventions can improve the well-being of healthcare workers and reduce burnout. The consistency and longevity of the reported effects vary from study to study [29,30,31]. Because none of these interventions was evaluated in the present study, and the design was cross-sectional, they should be understood as hypotheses for future evaluation rather than as strategies whose effectiveness this study can demonstrate.

### 4.8. Strengths and Limitations

Burnout was measured with a validated 20-item instrument, and all scores were computed from participants’ raw item responses rather than from pre-scored totals, which allowed the scoring and reverse-coding to be verified. The analysis used an adjusted regression framework with heteroscedasticity-consistent standard errors and considered demographic, health-related, and occupational characteristics together rather than in isolation.

Related to limitations, the cross-sectional design rules out conclusions about causality or temporal direction. Convenience sampling from two hospitals and the modest sample size limit generalizability, and the study population has highly specific characteristics. All participants worked full-time for more than 50 h per week, 87% worked 24-h shifts, and most held technical-institute qualifications and staff-nurse positions. The findings should therefore be interpreted within the context of these military hospitals and not extrapolated to civilian institutions, other healthcare settings, or differently organized health systems. Work–life balance satisfaction, physical activity, and chronic disease were self-reported. Work–life balance was captured by a single ordinal item that could not reflect the multidimensional nature of the construct and was modeled as a linear trend, although the unadjusted category means suggested that the gradient may not have been strictly linear. Common-method variance arising from the simultaneous self-report of burnout and work–life balance may also have inflated their association. Future studies should therefore employ validated multidimensional work–life balance instruments and test the linearity assumption through dummy-coded sensitivity analyses. The internal consistency of the occupational burnout domain fell below the conventional 0.70 threshold (α = 0.650), and the two-domain grouping of the BOSAS was not verified through factor analysis in the present data; domain-level results therefore warrant particular caution. Limited variability in shift patterns and weekly working hours may have reduced the capacity to detect associations with burnout. Finally, several subgroups were small: male nurses, participants aged 36–45 years, and nurses working 12-h shifts. Accordingly, these subgroup estimates should be interpreted with caution. The relatively small sample size may have limited statistical power for detecting modest associations. A further concern attaches to the way the data were gathered. Questionnaires were handed out and collected in person during working hours in the clinical units, and returns were checked for completeness on the spot. Participation was voluntary and nothing identifying was recorded, but within a military hospital hierarchy an arrangement of this kind can affect how voluntary participation feels, encourage socially desirable answers, and make respondents less willing to leave a sensitive item blank. The completeness of the returns should be judged against that. The study assessed the presence of any self-reported chronic disease without differentiating disease types or severity.

### 4.9. Implications for Nursing Management and Practice

For nursing managers, these findings suggest that work–life balance deserves a central place in burnout prevention efforts. Strategies that increase the predictability of schedules and strengthen protections around recovery time between shifts could ease the competing demands that work places on nurses’ personal lives. Nurses with chronic illnesses, along with early career nurses, may especially benefit from the provision of occupational health services and from early burnout screening. With the caveats set out above, the more plausible target for prevention is the organization of work as a whole rather than shift length on its own. That proposition has not been tested here and would need an intervention study to support it.

## 5. Conclusions

All nurses in this sample scored within the burnout range, most at moderate or severe levels. This partly reflects the classification thresholds of the instrument as well as the demanding work environment. Among the variables examined, work–life balance satisfaction showed the strongest association with burnout, chronic disease showed a borderline positive association, and shift duration was not independently associated; given the near-uniformity of shift patterns in this sample and the possible mediating role of work–life balance, however, no conclusion about the unimportance of shift length should be drawn. These cross-sectional associations cannot establish causality or direction. Subject to that caution, scheduling predictability, adequate recovery time, and protected work–life balance are the organizational levers most worth evaluating, alongside rather than instead of attention to shift duration. Longitudinal, multisite studies are needed to clarify these relationships and to test the effectiveness of preventive interventions.

## Figures and Tables

**Table 1:** Sociodemographic, work-related, and health characteristics of participants (N = 100).

Characteristic	Category	*n* (%)
Age, years	18–25	45 (45.0)
	26–35	49 (49.0)
	36–45	6 (6.0)
Gender	Male	14 (14.0)
	Female	86 (86.0)
Marital status	Single	52 (52.0)
	Married	47 (47.0)
	Divorced	1 (1.0)
Educational qualification	Nursing diploma	6 (6.0)
	Technical nursing institute	82 (82.0)
	Bachelor’s degree	10 (10.0)
	Master’s degree	2 (2.0)
Professional experience, years	0–5	40 (40.0)
	6–10	31 (31.0)
	11–15	18 (18.0)
	16–20	7 (7.0)
	≥21	4 (4.0)
Current position	Staff nurse	89 (89.0)
	Head nurse	11 (11.0)
Residence	Rural	53 (53.0)
	Urban	47 (47.0)
Shift duration	12-h shift	13 (13.0)
	24-h shift	87 (87.0)
Weekly working hours	≥51 h	100 (100.0)
Night shifts per month ^1^	0–5	4 (30.8)
	6–10	9 (69.2)
Employment status	Full-time	100 (100.0)
Chronic disease	Absent	79 (79.0)
	Present	21 (21.0)
Physical activity	Inactive	8 (8.0)
	Moderately active	70 (70.0)
	Active	22 (22.0)
Work–life balance satisfaction	Very dissatisfied	22 (22.0)
	Dissatisfied	21 (21.0)
	Neutral	26 (26.0)
	Satisfied	25 (25.0)
	Very satisfied	6 (6.0)

Note: ^1^ Night-shift frequency was recorded only for the 13 nurses working 12-h shifts, whose schedules included separate night duties; for nurses on 24-h shifts, night coverage was inherent to the shift itself, so the item did not apply. Percentages are calculated from that subgroup.

**Table 2:** BOSAS scores and burnout levels among participants (N = 100).

BOSAS/Domain	Number of Items	Possible Range	Observed Range	Mean ± SD	Cronbach’s α
Personal burnout	10	0–40	9–38	25.27 ± 6.54	0.773
Occupational burnout	10	0–40	9–37	21.45 ± 5.99	0.650
Total BOSAS score	20	0–80	21–69	46.72 ± 11.55	0.832
Burnout level		Score range		*n* (%)	
No burnout		0–20		0 (0.0)	
Mild burnout		21–40		30 (30.0)	
Moderate burnout		41–60		57 (57.0)	
Severe burnout		61–80		13 (13.0)	

**Table 3:** Unadjusted associations between participant characteristics and total burnout score (N = 100).

Characteristic	Category	*n*	Total BOSAS, Mean ± SD	*p* Value
Age, years	18–25	45	49.07 ± 10.61	**0.048**
	26–35	49	45.59 ± 12.09	
	36–45	6	38.33 ± 10.01	
Gender	Male	14	41.50 ± 12.09	0.089
	Female	86	47.57 ± 11.30	
Shift duration	12-h shift	13	51.69 ± 9.55	0.059
	24-h shift	87	45.98 ± 11.68	
Chronic disease	Absent	79	45.57 ± 11.85	**0.027**
	Present	21	51.05 ± 9.34	
Physical activity	Inactive	8	58.75 ± 7.42	**<0.001**
	Moderately active	70	47.51 ± 10.99	
	Active	22	39.82 ± 10.31	
Work–life balance satisfaction	Very dissatisfied	22	57.59 ± 10.31	**<0.001**
	Dissatisfied	21	50.62 ± 6.74	
	Neutral	26	42.12 ± 10.42	
	Satisfied	25	41.64 ± 9.40	
	Very satisfied	6	34.33 ± 6.09	

Note: *p* values were obtained from univariable linear regression models using HC3 robust standard errors. Overall Wald tests were used for age and physical activity. Work–life balance satisfaction was entered as an ordinal trend variable. Bold values indicate statistical significance at *p* < 0.05.

**Table 4:** Hierarchical multiple linear regression model summary for total burnout score (N = 100).

Model	Variables Entered	R^2^	Adjusted R^2^	ΔR^2^	F Change	*p* for Change
Model 1	Age and gender	0.094	0.066	—	—	—
Model 2	Model 1 + shift duration, chronic disease, and physical activity	0.268	0.212	0.174	5.46	<0.001
Model 3	Model 2 + work–life balance satisfaction	0.439	0.390	0.171	27.80	<0.001

Note: Model 1: F(3, 96) = 3.32, *p* = 0.023. Model 2: F(7, 92) = 4.81, *p* < 0.001. Final model: F(8, 91) = 8.91, *p* < 0.001.

**Table 5:** Multivariable linear regression analysis of factors associated with total burnout score (N = 100).

Variable	B	Robust SE	Standardized β	95% CI for B	*p* Value
Intercept	61.01	4.14	—	52.90 to 69.13	<0.001
Age, years					
18–25	Reference	—	—	—	—
26–35	−2.83	2.04	−0.123	−6.82 to 1.17	0.165
36–45	−10.14	4.91	−0.210	−19.77 to −0.50	0.039
Gender					
Male	Reference	—	—	—	—
Female	1.03	3.03	0.031	−4.92 to 6.97	0.735
Shift duration					
12-h shift	Reference	—	—	—	—
24-h shift	−2.74	2.34	−0.080	−7.33 to 1.84	0.241
Chronic disease					
Absent	Reference	—	—	—	—
Present	4.40	2.24	0.156	0.003 to 8.80	0.0499
Physical activity					
Inactive	Reference	—	—	—	—
Moderately active	−3.77	3.47	−0.151	−10.57 to 3.02	0.276
Active	−6.14	3.98	−0.221	−13.95 to 1.66	0.123
Work–life balance satisfaction ^1^	−4.49	0.94	−0.479	−6.33 to −2.66	<0.001

Model statistics: R^2^ = 0.439; adjusted R^2^ = 0.390; F(8, 91) = 8.91; *p* < 0.001. Note: B, unstandardized regression coefficient; SE, standard error; β, standardized regression coefficient; CI, confidence interval. HC3 robust standard errors were used. ^1^ Work–life balance satisfaction was coded from 0 = very dissatisfied to 4 = very satisfied; negative coefficients therefore indicate lower burnout with greater satisfaction. Average weekly working hours were excluded because all participants reported working at least 51 h per week; night-shift frequency was excluded because it was available for only 13 participants. Bold values indicate statistical significance at *p* < 0.05. For chronic disease, the exact HC3 robust *p* value was 0.0499 and is shown to four decimal places to avoid ambiguity from rounding to 0.050.

## Data Availability

The dataset generated and analysed during this study is fully anonymized and contains no identifying information. It has not been deposited in a public repository; it is available from the corresponding author on reasonable request.

## Supplementary Materials

The following supporting information can be downloaded at: https://www.mdpi.com/article/10.3390/healthcare14183015/s1, Supplementary Material S1: STROBE Statement.
