# Supplementary material for: Factors Associated with Burnout Among Nurses: The Roles of Shift Work Characteristics, Work–Life Balance Satisfaction, and Chronic Disease
Source: Healthcare (Basel). 2026 Sep 15;14(18):3015. doi: 10.3390/healthcare14183015 (PMC13606480; doi:10.3390/healthcare14183015)
Supplement: Supplementary file 1 [file healthcare-14-03015-s001.zip › healthcare-4511047-supplementary.pdf]

Supplementary Material S1. STROBE Statement

Checklist of items that should be included in reports of cross-sectional studies

Title: Factors Associated with Burnout Among Nurses: The Roles of Shift Work Characteristics, Work–Life Balance Satisfaction, and Chronic Disease.

Section and item numbers follow the revised manuscript. Where an item could not be met, the reason is stated rather than a section reference.

Item	Section/Topic	Recommendation	Reported in
1	Title and abstract	(a) Indicate the study's design with a commonly used term in the title or the abstract.	Abstract (Methods); Keywords
		(b) Provide in the abstract an informative and balanced summary of what was done and what was found.	Abstract
**Introduction**			
2	Background/rationale	Explain the scientific background and rationale for the investigation being reported.	Section 1, paragraphs 1–5
3	Objectives	State specific objectives, including any prespecified hypotheses.	Section 1, final paragraph
**Methods**			
4	Study design	Present key elements of study design early in the paper.	Section 2.1
5	Setting	Describe the setting, locations, and relevant dates, including periods of recruitment, exposure, follow-up, and data collection.	Sections 2.1, 2.6
6	Participants	(a) Give the eligibility criteria, and the sources and methods of selection of participants.	Section 2.2
7	Variables	Clearly define all outcomes, exposures, predictors, potential confounders, and effect modifiers. Give diagnostic criteria, if applicable.	Sections 2.4, 2.7
8	Data sources/ measurement	For each variable of interest, give sources of data and details of methods of assessment. Describe comparability of assessment methods if there is more than one group.	Sections 2.4, 2.5
9	Bias	Describe any efforts to address potential sources of bias.	Sections 2.6, 4.8
10	Study size	Explain how the study size was arrived at.	Section 2.3
11	Quantitative variables	Explain how quantitative variables were handled in the analyses. If applicable, describe which groupings were chosen and why.	Sections 2.4, 2.7
12	Statistical methods	(a) Describe all statistical methods, including those used to control for confounding.	Section 2.7
		(b) Describe any methods used to examine subgroups and interactions.	Section 2.7 (dummy-coded categories); no interaction terms were tested
		(c) Explain how missing data were addressed.	Sections 2.2, 2.6: all returned questionnaires were complete and no variable analysed had missing values, so no imputation was required

Item	Section/Topic	Recommendation	Reported in
		(d) If applicable, describe analytical methods taking account of sampling strategy.	Not applicable: convenience sampling without weighting (Section 2.2)
		(e) Describe any sensitivity analyses.	Sections 2.7, 3.5
**Results**			
13	Participants	(a) Report numbers of individuals at each stage of the study.	Section 2.2: 100 nurses present during data collection visits agreed to participate and were analysed
		(b) Give reasons for non-participation at each stage.	Not available. Section 2.2 states that the study records do not document the number approached or the number who declined
		(c) Consider use of a flow diagram.	Not provided; the stage-by-stage counts required for a flow diagram were not recorded (Section 2.2)
14	Descriptive data	(a) Give characteristics of study participants and information on exposures and potential confounders.	Section 3.1; Table 1
		(b) Indicate the number of participants with missing data for each variable of interest.	None for any analysed variable; see item 12(c). Night-shift frequency was not analysed, as it applied only to the 13 nurses on 12-hour shifts (Section 2.7; note to Table 1)
15	Outcome data	Report numbers of outcome events or summary measures.	Section 3.2; Table 2
16	Main results	(a) Give unadjusted estimates and, if applicable, confounder-adjusted estimates and their precision. Make clear which confounders were adjusted for and why they were included.	Sections 3.3, 3.4, 3.5; Tables 3, 4, 5. Adjustment set and rationale in Sections 2.3 and 2.7
		(b) Report category boundaries when continuous variables were categorized.	Section 2.4 (BOSAS burnout bands); Table 1 (age, experience, and work–life balance categories)
		(c) If relevant, consider translating estimates of relative risk into absolute risk for a meaningful time period.	Not applicable: the outcome is a continuous burnout score, and coefficients are reported on the original scale
17	Other analyses	Report other analyses done, such as analyses of subgroups and interactions, and sensitivity analyses.	Section 3.5: dummy-coded sensitivity analysis for work–life balance satisfaction
**Discussion**			
18	Key results	Summarise key results with reference to study objectives.	Section 4.1

Item	Section/Topic	Recommendation	Reported in
19	Limitations	Discuss limitations of the study, taking into account sources of potential bias or imprecision. Discuss both direction and magnitude of any potential bias.	Section 4.8; see also Sections 4.2, 4.3, 4.5
20	Interpretation	Give a cautious overall interpretation of results considering objectives, limitations, multiplicity of analyses, results from similar studies, and other relevant evidence.	Sections 4.3–4.7, 4.9; Section 5
21	Generalisability	Discuss the generalisability (external validity) of the study results.	Section 4.8
**Other information**			
22	Funding	Give the source of funding and the role of the funders for the present study and, if applicable, for the original study on which the present article is based.	Funding statement; Acknowledgments

Note. Items 13(b) and 13(c) could not be completed. The study records do not document the size of the eligible nursing workforce, the number of nurses approached, or the number who declined, so the stage-by-stage counts required for a participant flow diagram are unavailable. This limitation is stated in Section 2.2 of the revised manuscript and is discussed in Section 4.8.

An Explanation and Elaboration article discusses each checklist item and gives methodological background and published examples of transparent reporting. The STROBE checklist is best used in conjunction with this article, which is freely available on the websites of PLoS Medicine, Annals of Internal Medicine, and Epidemiology. Information on the STROBE Initiative is available at www.strobe-statement.org.
